# Severity of maternal infection and perinatal outcomes during periods of SARS-CoV-2 wildtype, alpha, and delta variant dominance in the UK: prospective cohort study

**DOI:** 10.1136/bmjmed-2021-000053

**Published:** 2022-02-28

**Authors:** Nicola Vousden, Rema Ramakrishnan, Kathryn Bunch, Eddie Morris, Nigel A B Simpson, Christopher Gale, Patrick O'Brien, Maria Quigley, Peter Brocklehurst, Jennifer J Kurinczuk, Marian Knight

**Affiliations:** 1 National Perinatal Epidemiology Unit, Nuffield Department of Population Health, University of Oxford, Oxford, UK; 2 Royal College of Obstetricians and Gynaecologists, London, UK; 3 Department of Women’s & Children’s Health, University of Leeds, Leeds, UK; 4 School of Public Health, Faculty of Medicine, Imperial College London, London, UK; 5 Institute for Women's Health, University College London, London, UK; 6 Birmingham Clinical Trials Unit, University of Birmingham, Birmingham, West Midlands, UK

**Keywords:** COVID-19, pregnancy complications, neonatology

## Abstract

**Objective:**

To compare the severity of maternal infection and perinatal outcomes during periods in which wildtype, alpha variant, and delta variant of SARS-CoV-2 were dominant in the UK.

**Design:**

Prospective cohort study.

**Setting:**

194 obstetric units across the UK, during the following periods: between 1 March and 30 November 2020 (wildtype dominance), between 1 December 2020 and 15 May 2021 (alpha variant dominance), and between 16 May and 31 October 2021 (delta variant dominance).

**Participants:**

4436 pregnant women admitted to hospital with covid-19 related symptoms.

**Main outcome measures:**

Moderate to severe maternal SARS-CoV-2 infection (indicated by any of the following: oxygen saturation <95% on admission, need for oxygen treatment, evidence of pneumonia on imaging, admission to intensive care, or maternal death), and pregnancy and perinatal outcomes (including mode and gestation of birth, stillbirth, live birth, admission to neonatal intensive care, and neonatal death).

**Results:**

1387, 1613, and 1436 pregnant women were admitted to hospital with covid-19 related symptoms during the wildtype, alpha, and delta dominance periods, respectively; of these women, 340, 585, and 614 had moderate to severe infection, respectively. The proportion of pregnant women admitted with moderate to severe infection increased during the subsequent alpha and delta dominance periods, compared with the wildtype dominance period (wildtype 24.5% *v* alpha 36.2% (adjusted odds ratio 1.98, 95% confidence interval 1.66% to 2.37%); wildtype 24.5% *v* delta 42.8% (2.66, 2.21 to 3.20)). Compared with the wildtype dominance period, women admitted during the alpha dominance period were significantly more likely to have pneumonia, require respiratory support, and be admitted to intensive care; these three risks were even greater during the delta dominance period (wildtype *v* delta: pneumonia, adjusted odds ratio 2.52, 95% confidence interval 2.06 to 3.09; respiratory support, 1.90, 1.52 to 2.37; and intensive care, 2.71, 2.06 to 3.56). Of 1761 women whose vaccination status was known, 38 (2.2%) had one dose and 16 (1%) had two doses before their diagnosis (of whom 14 (88%) had mild infection). The proportion of women receiving drug treatment for SARS-CoV-2 management was low, but did increase between the wildtype dominance period and the alpha and delta dominance periods (10.4% wildtype *v* 14.9% alpha (2.74, 2.08 to 3.60); 10.4% wildtype *v* 13.6% delta (2.54, 1.90 to 3.38)).

**Conclusions:**

While limited by the absence of variant sequencing data, these findings suggest that during the periods when the alpha and delta variants of SARS-CoV-2 were dominant, covid-19 was associated with more severe maternal infection and worse pregnancy outcomes than during the wildtype dominance period. Most women admitted with SARS-CoV-2 related symptoms were unvaccinated. Urgent action to prioritise vaccine uptake in pregnancy is essential.

**Study registration:**

ISRCTN40092247.

**What is already known on this topic**

- In non-pregnant adults, growing evidence suggests that infection with the SARS-CoV-2 alpha and delta variants of concern increases the risk of hospital admission
- Data on the effect of SARS-CoV-2 variants during pregnancy is limited to three descriptive single hospital studies and a review of maternal mortality registries in Brazil
- Robust evidence is needed on the impact of SARS-CoV-2 infection in pregnancy during periods when wildtype, alpha, and delta variants were predominant on the severity of maternal infection or perinatal outcomes

**What this study adds**

- Pregnant women admitted to UK hospitals during the period of SARS-CoV-2 alpha and delta variance dominance were at increased risk of moderate to severe infection compared with when the original wildtype was dominant
- Effective treatments were used in only a minority of women, even among those who were critically unwell
- Most women admitted with symptoms of SARS-CoV-2 were unvaccinated

**How this study might affect research, practice, or policy**

- Increased risk of severe disease in pregnancy emphasises the importance of ensuring that all pregnant women receive appropriate, evidence based, medical and respiratory treatments for covid-19 and are not denied treatment simply on the basis of their pregnancy
- As new variants of concern emerge, assessment of the impact of covid-19 on pregnant women will remain important because, as indicated by this study's findings, the outcomes for both women and their offspring could be different

## Introduction

In 2020, the World Health Organization's living systematic review concluded that SARS-CoV-2 infection during pregnancy was associated with an increased risk of admission to intensive care for mothers, preterm birth, and admission for neonatal care for infants.^1^ Included studies predominantly contained data from the US and China and were conducted in the first six months of the pandemic, before the spread of new variants.

**Figure 1:**
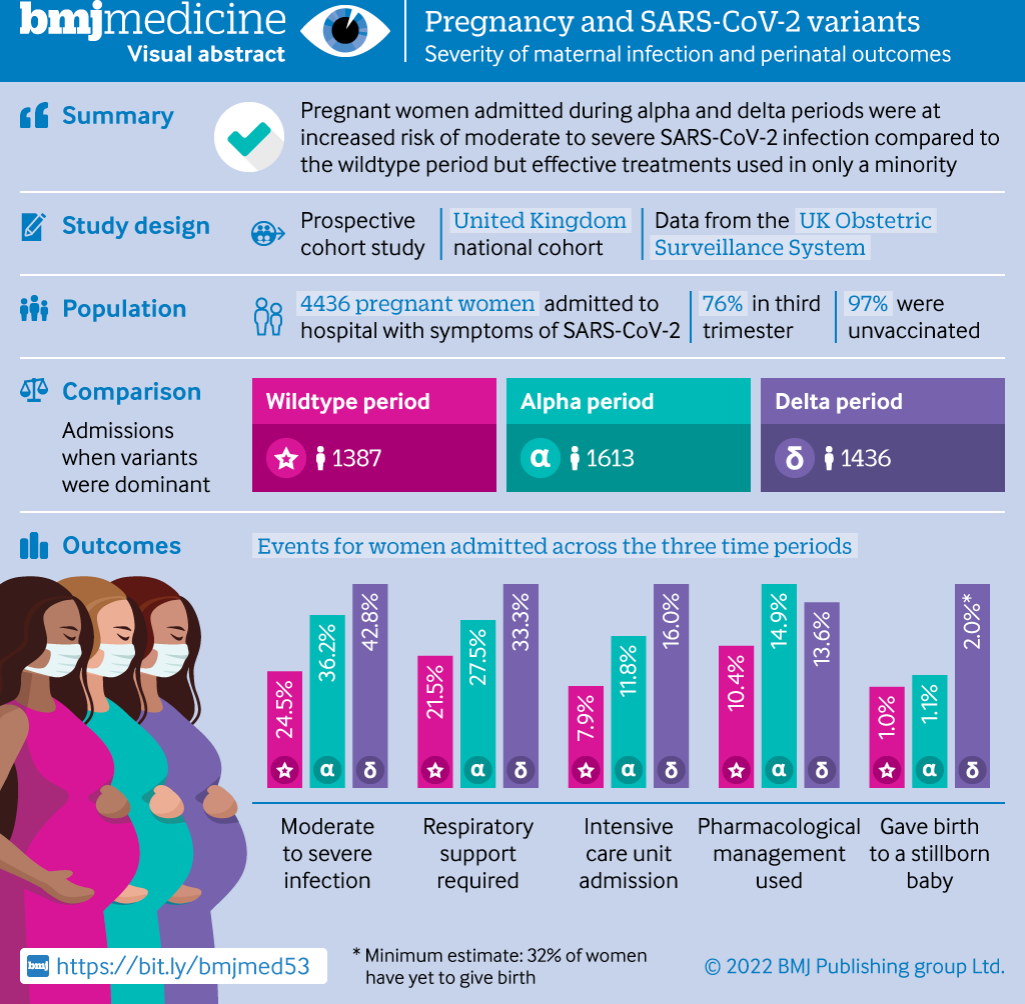
Visual abstract

In the UK, a new variant of SARS-CoV-2 (B.1.1.7, alpha variant of concern) was initially reported in southeast England in September 2020 and then circulated at very low levels in the population until mid-November 2020 when it then became dominant.^2^ The alpha variant was then succeeded by the delta variant (B.1.617.2), which became dominant in the UK in late May 2021.^3^ Growing evidence indicates that in the non-pregnant population, the alpha variant might be associated with an increased risk of hospital admission and mortality compared with other lineages.^4^ Most recently, data from a Scottish national cohort showed that infection with the delta variant roughly doubled the risk of hospital admission in the general population, compared with infection with the alpha variant.^5^ However, very few published studies have explored the impact of different SARS-CoV-2 variants or variant periods on pregnancy and perinatal outcomes. A single centre study from the UK reported a substantial increase in peripartum referrals for extracorporeal membrane oxygenation during the second wave of the pandemic, when the alpha variant became dominant (n=19 *v* n=4 in the wildtype dominance period).^6^ These findings accorded with those of a national registry of patients admitted to intensive care, which reported an increase in the number of pregnant or recently pregnant women admitted in the second wave compared with the first wave.^7^ However, these reports were limited by the absence of a comparator, meaning it was not possible to determine whether this difference was a result of changing variant periods as opposed to an increasing total number of infected women.

To our knowledge, only two further publications have explored the potential impact of different SARS-CoV-2 variant periods in pregnancy. A retrospective cohort from a single centre in India concluded that pregnant women admitted to hospital during the dominant second wave of the delta variant (n=387) had higher rates of admission to intensive care or high dependency units (11.6 *v* 2.4%) and case fatality (5.7 *v* 0.7%) than those admitted during the first wave (n=1143).^8^ This difference accords with a review of 803 maternal deaths with SARS-CoV-2 in Brazil, where a substantially higher case fatality rate was reported in 2021 (P.1, gamma variant) than in 2020 (15.6% *v* 7.4%).^9^ These preliminary studies suggest an urgent need for robust national data on the impact of new variants or periods in which new variants were predominant on maternal and perinatal outcomes in order to inform policy. The primary aim of this study was therefore to compare the impact of SARS-CoV-2 infection on severity of maternal infection and perinatal outcomes across three time periods in which the original wildtype, alpha variant, and delta variant of SARS-CoV-2 were dominant. This analysis was completed before the omicron variant emerged. The study was registered with ISRCTN40092247, and the protocol is available at https://wwwnpeuoxacuk/ukoss/current-surveillance/covid-19-in-pregnancy. For the visual abstract of this paper, see figure 1.

## Methods

### Data sources

A national, prospective observational cohort study was conducted using the UK Obstetric Surveillance System (UKOSS).^10^ UKOSS is a research platform that was established in 2005. All 194 hospitals in the UK with a consultant led maternity unit collect population based information about specific severe pregnancy complications. Nominated reporting clinicians, facilitated by research midwives and nurses from the UK’s National Institute of Health Research Clinical Research Network, sent notification of all pregnant women admitted to their hospital with confirmed SARS-CoV-2 infection to UKOSS. In addition to receipt of real time notifications, hospitals that did not notify any admissions were confirmed to have zero admissions. Reporters who had sent notifications but not returned data received email reminders by the UKOSS team. Hospital admission was defined as an overnight hospital stay, or longer, for any cause, or admission of any duration to give birth. Women were regarded as having confirmed SARS-CoV-2 if they were admitted during pregnancy and they had a positive test in the seven days prior to admission or during their admission. Women who only had a positive test during admission which was more than two days after giving birth were excluded. Women not meeting this definition, and those without any symptoms of SARS-CoV-2 infection, were excluded (online supplemental figure 1). Information on women who died, or who had stillbirths or neonatal deaths, was cross checked with data from the organisation responsible for maternal and perinatal death surveillance in the UK (MBRRACE-UK).^11^

10.1136/bmjmed-2021-000053.supp1 Supplementary data

### Measures

The primary outcome was a composite indicating moderate to severe SARS-CoV-2 infection that was based on the WHO criteria of COVID-19 disease severity.^12^ Women with any one of the following factors were classified as having moderate to severe disease: oxygen saturation <95% on admission, need for oxygen treatment, evidence of pneumonia on imaging, admission to intensive care, or maternal death. Each of those components was also analysed separately, as were pregnancy and perinatal outcomes including mode and gestation of birth, stillbirth, live birth, admission to neonatal intensive care, and neonatal death.

As individual level SARS-CoV-2 variant data were not recorded in medical records, the outcomes were compared across three proxy groups according to the time period in which the original wildtype, alpha variant, and delta variant were the dominant circulating strain in the UK. The original wildtype period included women admitted to hospital from 1 March to 30 November 2020, the alpha variant period from 1 December 2020 to 15 May 2021, and the delta variant period from 16 May 2021 to 31 October 2021. We chose cut-off dates for the delta period using data on variant sequencing from Public Health England to identify the week when this variant first contributed more than 50% of covid-19 infections nationally.^3^ Since genomic data on the variant were less widely available at the start of the pandemic, Public Health England modelled proxy data and reported that the alpha variant reflected the substantial majority of infections across all areas of England during December 2020; therefore, 1 December was used as an estimated cut-off date.^13^ Analysis of drug treatments was restricted to women admitted on or after 1 July 2020, when national guidance on drug treatments was available.

### Statistical analysis

Statistical analyses were performed using Stata version 15 (StataCorp, TX). Numbers and proportions are presented with 95% confidence intervals. If data were missing, proportions were presented out of number of infections known. We estimated odds ratios (95% confidence intervals) using multilevel binary (dichotomous outcomes) and multinomial logistic regression (categorical outcomes) and quantile regression (continuous gestational age) models to account for clustering of women within hospitals.

The hypothesised associations between SARS-CoV-2 variant period and severity of infection were identified by use of directed acyclic graphs, created with DAGitty.net^14 15^ (online supplemental figure 2). These relations were informed by associations identified in the literature and underlying theory. The minimum adjustment set to control for confounding bias was personal characteristics (age, ethnic origin, body mass index, and employment) and vaccine status. However, data were not sufficient enough to include vaccine status as a covariate (because vaccine status data were only collected from 1 February 2021) and therefore, based on the directed acyclic graph, we had to also include pre-existing medical conditions (asthma, cardiac disease, diabetes, or hypertension) to block a further potential biasing pathway (online supplemental figure 2).^16^ These conditions were included in the model as a combined covariate if any of the conditions were identified. In the absence of data sparsity or multicollinearity (highest Spearman correlation coefficient of 0.19), all prespecified covariates as identified by the directed acyclic graph were included. After testing for departure from linearity using likelihood ratio testing, body mass index was included as ordered categorical variables and grouped into clinically meaningful categories; and age and ethnic origin were included as binary covariates to avoid data sparsity. We did sensitivity analyses including body mass index and age as continuous variables using restricted cubic spline with three knots each. For body mass index, these knots were located at values of 21.3, 27.3, and 37.6, and for age, at 23 years, 31 years, and 38 years. Potential effect modifiers were identified a priori as the covariates identified in the directed acyclic graph, in addition to parity and trimester of pregnancy at time of infection. Plausible interactions were tested by the addition of interaction terms and subsequent likelihood ratio testing on removal, with P<0.01 considered as evidence of significant interaction to account for multiple testing. No interaction terms were included in the model. Because the amounts of missing data were small, we used a complete case analysis approach. We conducted further sensitivity analyses using multiple imputation (m=15) by chained equations using fully conditional specifications to account for missing values. In this national observational study, the study sample size was governed by the disease incidence, thus no formal power calculation was carried out.

### Patient and public involvement

Patients and public were part of the UKOSS steering committee and were involved in study oversight but not in the design, reporting, conduct, or dissemination of this study. As this study used anonymous data, dissemination to women whose data were included is not possible. Results will be disseminated to public communities through the websites and social media channels of the National Perinatal Epidemiology Unit and the Nuffield Department of Population Health, University of Oxford.

## Results

**Figure 2:**
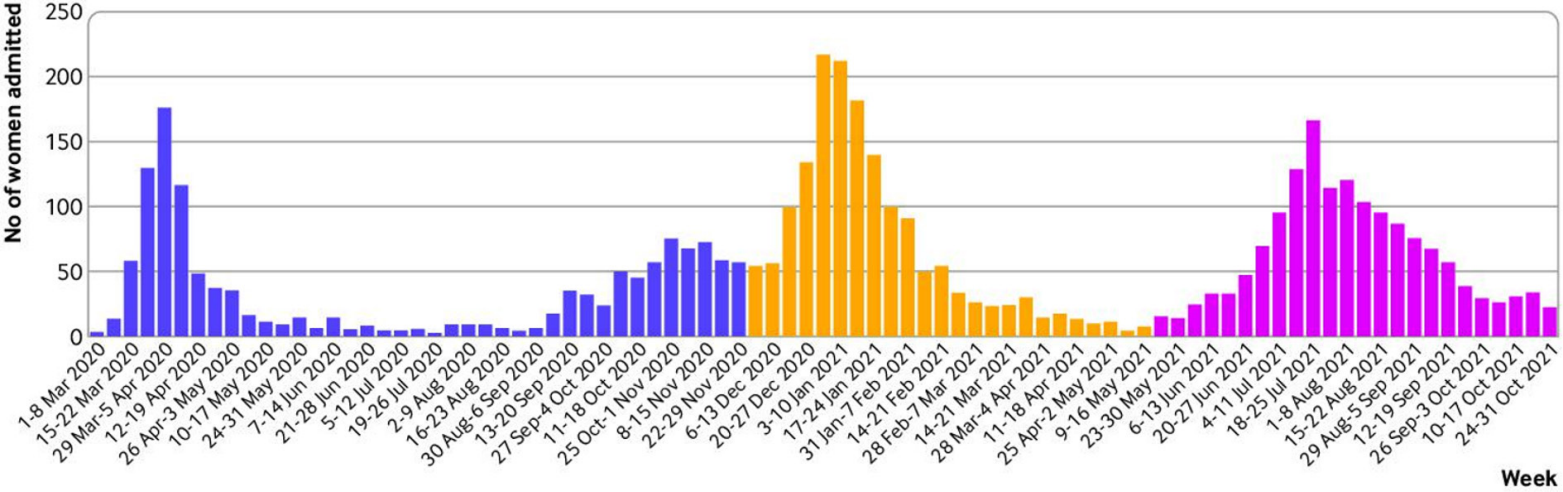
Admissions of pregnant women with symptoms of confirmed SARS-CoV-2 to UK hospitals during dominance periods of original wildtype (1 March to 30 November 2020), alpha variant (1 December 2020 to 15 May 2021), and delta variant (16 May to 31 October 2021)

In total, 4436 women were admitted to hospital from 186 obstetric units across the UK with symptoms of confirmed SARS-CoV-2 infection between 1 March 2020 and 31 October 2021. Most infections occurred during the second wave when the alpha variant was dominant (figure 2). Of those women whose primary reason for admission was known (78.6%, n=3486), half (49.2%, n=1716) were admitted for covid-19, 25.7% (n=895) for labour and birth, and 25.1% (n=874) for other obstetric reasons.

The characteristics of each group are described in table 1. Across all three periods, just over a quarter of women were aged 35 years or over (26.1% (n=371; delta) *v* 28.3% (n=456; alpha) and 29.8% (n=413; wildtype)). In the delta period, 71% (n=1017) of women reported that they or their partner were in paid employment compared with nearly 80% in the alpha and wildtype periods (75.0% (n=1210) and 79.7% (n=1106), respectively). Across all three periods, most women included in the study were overweight or obese. The proportion of women admitted in the delta and alpha periods with one or more pre-existing medical conditions was 13.5% (n=194) and 13.7% (n=221), respectively, compared with 11.9% (n=165) of those admitted in the wildtype period. The most common gestation at admission was term across all three time periods.

**Table 1:** Characteristics of pregnant women admitted to UK hospitals with SARS-CoV-2 related symptoms during periods in which the original wildtype, alpha variant, and delta variant were dominant

Characteristic	Wildtype period (n=1387)	Alpha variant period (n=1613)	Delta variant period (n=1436)
Age (years)			
<20	20 (1.4)	18 (1.1)	36 (2.5)
20-34	953 (68.8)	1137 (70.6)	1015 (71.4)
≥35	413 (29.8)	456 (28.3)	371 (26.1)
Missing data	1	2	13
Body mass index			
Underweight (<18.5)	21 (1.6)	16 (1.0)	21 (1.5)
Normal (18.5 to <25)	439 (32.8)	517 (33.6)	434 (31.9)
Overweight (25 to <30)	432 (32.3)	454 (29.5)	442 (32.4)
Obese (≥30)	446 (33.3)	553 (35.9)	465 (34.2)
Missing data	49	73	74
Either woman or partner in paid work	1106 (79.7)	1210 (75.0)	1017 (70.8)
Ethnic group			
White	675 (49.7)	902 (57.3)	965 (69.3)
Asian	409 (30.1)	400 (25.4)	208 (14.9)
Black	173 (12.7)	163 (10.4)	128 (9.2)
Chinese/other	71 (5.2)	66 (4.2)	59 (4.3)
Mixed	31 (2.3)	43 (2.7)	32 (2.3)
Missing data	28	39	44
Current smoking	97 (7.3)	137 (8.7)	168 (12.2)
Missing data	62	42	55
Pre-existing medical conditions	165 (11.9)	221 (13.7)	194 (13.5)
Asthma	91 (6.6)	163 (10.1)	149 (10.4)
Hypertension	39 (2.8)	30 (1.9)	17 (1.2)
Cardiac disease	20 (1.4)	18 (1.1)	15 (1.0)
Diabetes	35 (2.5)	27 (1.7)	21 (1.5)
Gestational diabetes	143 (10.3)	187 (11.6)	109 (7.6)
Multiparous	828 (60.1)	1065 (66.5)	944 (67.0)
Missing	9	12	26
Multiple pregnancy	36 (2.6)	35 (2.2)	35 (2.4)
Gestation at admission (weeks+days)			
<22	147 (10.6)	178 (11.2)	130 (9.2)
22-27^+6^	155 (11.2)	214 (13.4)	214 (15.2)
28-33^+6^	295 (21.4)	354 (22.2)	341 (24.1)
34-36^+6^	217 (15.7)	258 (16.2)	257 (18.2)
≥37	567 (41.1)	591 (37.1)	473 (33.4)
Missing data	6	18	24

Data are number (%) of women.

Vaccination status was obtained from 1 February 2021. Of 1761 pregnant women admitted with symptomatic SARS-CoV-2 and known vaccination status (n=24 during the wildtype period, n=557 during the alpha period, and n=1180 during the delta period), 97% were unvaccinated at the time of covid-19. Of the 38 (2.2%) women who had one vaccine dose before positive virology, 23 (60.5%) had mild infection; of 16 (1%) women who had two doses before their diagnosis, 14 (87.5%) had mild infection. Of the 17 women with moderate to severe infection who had been previously vaccinated, the most common characteristic indicating moderate to severe infection was requiring non-invasive ventilation (n=9).

Overall, 34.7% (n=1539) of women admitted with covid-19 related symptoms during the wildtype period had at least one marker of moderate to severe infection. The odds of moderate or severe infection significantly increased twofold during the alpha period (adjusted odds ratio 1.98, 95% confidence 1.66 to 2.37), and more than 2.5-fold during the delta period, when nearly half (42.8%) of women had moderate to severe infection (2.66, 2.21 to 3.20; table 2). A total of 22 maternal deaths in women with covid-19 were recorded: 10 during the wildtype period, six during the alpha period, and six during the delta period (table 2), noting that some women remained very ill in intensive care at the time of this analysis. After adjustment, women admitted during the alpha period were significantly more likely to require admission to intensive care than those admitted during the wildtype period (11.8% *v* 7.9%; 1.82, 1.38 to 2.39) and the risk increased further during the delta period (16.0% *v* 7.9%; 2.71, 2.06 to 3.56). We saw no material differences in the odds ratios obtained after multiple imputation for missing data (online supplemental tables S1–3) or after adjustment for age and body mass index using the cubic spline approach (online supplemental tables S4–6); therefore, complete case models adjusting for age and body mass index as categorical variables are presented from now on.

10.1136/bmjmed-2021-000053.supp2 Supplementary data

**Table 2:** Respiratory and medical support of pregnant women admitted to UK hospitals with SARS-CoV-2 related symptoms during periods in which the original wildtype, alpha variant, and delta variant were dominant

	Wildtype period (n=1387)	Alpha period (n=1613)	Delta period (n=1436)	Alpha *v* wildtype period		Delta *v* wildtype period	
				Odds ratio (95% CI)	Adjusted odds ratio (95% CI)*	Odds ratio (95% CI)	Adjusted odds ratio (95% CI)*
Composite indicator of moderate to severe infection	340 (24.5)	585 (36.2)	614 (42.8)	1.92 (1.63 to 2.27)	1.98 (1.66 to 2.37)	2.33 (1.97 to 2.77)	2.66 (2.21 to 3.20)
Oxygen saturation measured on admission (yes)	508 (36.6)	1118 (69.3)	1120 (78.0)	5.30 (4.42 to 6.35)	5.34 (4.45 to 6.41)	7.28 (5.97 to 8.88)	7.52 (6.14 to 9.21)
Oxygen saturation <95%	49 (9.7)	173 (15.5)	160 (14.3)	1.67 (1.18 to 2.36)	1.63 (1.14 to 2.34)	1.53 (1.08 to 2.18)	1.60 (1.10 to 2.31)
Evidence of pneumonia on imaging	264 (19.0)	445 (28.1)	466 (32.5)	1.81 (1.51 to 2.18)	1.86 (1.53 to 2.26)	2.20 (1.82 to 2.65)	2.52 (2.06 to 3.09)
Respiratory support required	186 (21.5)	482 (27.5)	458 (33.3)	1.48 (1.21 to 1.81)	1.43 (1.15 to 1.77)	1.82 (1.48 to 2.24)	1.90 (1.52 to 2.37)
Non-invasive oxygen (nasal canulae, mask, or non-rebreathe mask at <15 L/min)	89 (49.7)	255 (58.8)	236 (54.8)	Reference	Reference	Reference	Reference
High flow oxygen (>15 L/min) or continuous positive airway pressure	35 (19.6)	77 (17.7)	103 (23.9)	0.74 (0.46 to 1.20)	0.81 (0.49 to 1.34)	1.09 (0.68 to 1.75)	1.14 (0.69 to 1.89)
Invasive ventilation or extracorporeal membrane oxygenation	55 (30.7)	102 (23.5)	92 (21.4)	0.63 (0.41 to 0.96)	0.74 (0.47 to 1.16)	0.62 (0.40 to 0.95)	0.74 (0.46 to 1.18)
Level not known	7	11	27	—	—	—	—
Intensive care admission	109 (7.9)	190 (11.8)	230 (16.0)	1.63 (1.27 to 2.10)	1.82 (1.38 to 2.39)	2.26 (1.76 to 2.90)	2.71 (2.06 to 3.56)
Maternal death	10 (0.7)	6 (0.4)	6 (0.4)	Not compared	Not compared	Not compared	Not compared
Pharmacological management total†‡	70 (10.4)	240 (14.9)	195 (13.6)	2.57 (1.98 to 3.33)	2.74 (2.08 to 3.60)	2.33 (1.77 to 3.05)	2.54 (1.90 to 3.38)
Antivirals†	29 (4.3)	34 (2.1)	23 (1.6)	Not compared	Not compared	Not compared	Not compared
Tocilizumab†	0	24 (1.5)	36 (2.5)	Not compared	Not compared	Not compared	Not compared
Steroids for maternal indication†	51 (7.6)	205 (12.7)	172 (12.0)	Not compared	Not compared	Not compared	Not compared
Regeneron monoclonal antibodies†	0	6 (0.4)	0	Not compared	Not compared	Not compared	Not compared
Recruited to the RECOVERY trial	21 (1.5)	85 (5.3)	10 (0.7)	Not compared	Not compared	Not compared	Not compared
Steroids for fetal lung maturation	245 (17.7)	311 (19.3)	259 (18.0)	Not compared	Not compared	Not compared	Not compared

Data are number (%) of women.

*Adjusted for age, ethnic origin, body mass index, employment, and presence of one or more pre-existing relevant medical comorbidity.

†Wildtype period, n=672; restricted to women admitted on or after 1 July 2020 when guidance on management was available from the Royal College of Obstetrics and Gynaecologists.

‡Any of the listed drugs given for medical management of SARS-CoV-2.

Women admitted during the alpha period had a nearly twofold increased risk, and during the delta period a 2.5-fold increased risk, of SARS-CoV-2 pneumonia confirmed on imaging compared with those admitted in the wildtype period (28.1% alpha *v* 19.0% wildtype (adjusted odds ratio 1.86, 95% confidence interval 1.53 to 2.26); 32.5% delta *v* 19.0% wildtype (2.52, 2.06 to 3.09); table 2). Need for respiratory support similarly increased, with a third of women requiring respiratory support during the delta period (21.5% wildtype *v* 27.5% alpha (1.43, 1.15 to 1.77); 21.5% wildtype *v* 33.3% delta (1.90, 1.52 to 2.37)). Although not significant, there also a suggestion of reduced use of invasive ventilation or extracorporeal membrane oxygenation over time (30.7% wildtype *v* 23.5% alpha (0.74, 0.47 to 1.16); 30.7% wildtype *v* 21.4% delta (0.74, 0.46 to 1.18)).

The proportion of women who received any drug treatment for covid-19 (antivirals, tocilizumab, maternal steroids, or monoclonal antibodies) was small, but did increase between the wildtype period and the alpha and delta periods (10.4% wildtype *v* 14.9% alpha (adjusted odds ratio 2.74, 95% confidence interval 2.08 to 3.60); 10.4% wildtype *v* 13.6% delta (2.54, 1.90 to 3.38)). Across the whole study period, a higher proportion of women admitted to intensive care received any drug treatment for covid-19 (39.3%, n=208) than those not admitted to intensive care (8.2%, n=319), although this proportion was still small: 11.0% (n=58) received antivirals, 9.8% (n=52) received tocilizumab, 28.4% (n=150) received maternal steroids, and 0.4% (n=2) received monoclonal antibodies.

Of those pregnant women with complete outcome information (97.8% during the wildtype period, 95.8% during the alpha period, and 67.3% during the delta period), the median gestation at birth was the same across the wildtype and alpha periods (table 3). Births between 22 and <28 weeks’ gestation occurred for 2.1% (n=32) of women admitted during the alpha period, compared with 0.9% (n=13) of those admitted in the wildtype period (adjusted odds ratio 2.38, 95% confidence interval 1.13 to 5.00). Births between 28 and <34 weeks’ gestation occurred for 7.8% (n=119) of women admitted during the alpha period compared with 6.2% (n=83) of those admitted during the wildtype period (1.37, 1.00 to 1.87). As anticipated from the timing of this analysis, fewer pregnancies were completed in the delta period. Therefore, we did not formally compare the proportion of preterm births, mode of birth, and expedited delivery between wildtype and delta periods, because those women who were admitted during the delta period and had given birth by the time of analysis would be expected to be more likely to be preterm and expedited.

**Table 3:** Pregnancy outcomes for women admitted to UK hospitals with SARS-CoV-2 related symptoms during periods in which the original wildtype, alpha variant, and delta variant were dominant

Pregnancy outcomes	Wildtype period (n=1387)	Alpha period (n=1613)	Delta period (n=1436)	Alpha *v* wildtype period	
				Odds ratio (95% CI)	Adjusted odds ratio (95% CI)*
Pregnancy outcome known	1356 (97.8)	1545 (95.8)	967 (67.3)	Not compared	Not compared
Pregnancy loss	33 (2.4)	26 (1.7)	18 (1.9)	Not compared	Not compared
Birth	1323 (95.4)	1519 (94.2)	949 (66.1)	Not compared	Not compared
Gestation at birth (weeks+days)					
<22	29 (2.2)	18 (1.2)	13 (1.3)	Not compared	Not compared
22-27^+6^	13 (0.9)	32 (2.1)	20 (2.1)	2.11 (1.07 to 4.18)	2.38 (1.13 to 5.00)
28-34^+6^	83 (6.2)	119 (7.8)	95 (9.8)	1.30 (0.97 to 1.74)	1.37 (1.00 to 1.87)
34-36^+6^	148 (11.1)	173 (11.4)	149 (15.7)	1.07 (0.85 to 1.34)	1.06 (0.84 to 1.34)
37 or more	1065 (79.6)	1180 (77.5)	675 (69.8)	Reference	Reference
Missing data	18	23	15	—	—
Median (interquartile range)‡	39 (37-40)	39 (37-40)	39 (37-40)	−0.14 (−0.33 to 0.05)	−0.14 (−0.34 to 0.05)
Delivery expedited due to covid-19†	85 (11.9)	183 (12.7)	174 (20.8)	1.08 (0.82 to 1.42)	1.06 (0.79 to 1.43)
Missing data	610	79	111	—	—
Mode of birth†					
Pre-labour caesarean	416 (31.7)	551 (36.9)	376 (40.2)	1.21 (1.02 to 1.45)	1.17 (0.97 to 1.40)
Caesarean after labour onset	200 (15.2)	208 (13.9)	102 (10.9)	0.95 (0.76 to 1.20)	0.91 (0.72 to 1.15)
Operative vaginal	147 (11.2)	135 (9.0)	82 (8.8)	0.84 (0.65 to 1.09)	0.81 (0.62 to 1.06)
Unassisted vaginal	549 (41.8)	600 (40.2)	375 (40.1)	Reference	Reference
Missing data	11	25	14	—	—

Data are number (%) of women. Reference=reference group against which other categories were compared; wildtype to delta comparison purposely not undertaken owing to differing availability of pregnancy outcome data.

*Adjusted for age, ethnic origin, body mass index, employment, and presence of one or more pre-existing relevant medical comorbidity.

†Excluding pregnancy loss from denominator.

‡Quantile (median) regression using bootstrap sampling.

While most babies born to mothers included in this study were live born, 3.0% (n=29) of babies born to those women admitted to hospital with covid-19 related symptoms in the delta period were stillborn compared with 1.0% (n=13) born to women admitted in the wildtype period. These groups were not formally compared owing to the differences in availability of pregnancy outcome data for this period; however, if all babies who were yet to be born were liveborn, there would be a minimum stillbirth rate of 2.0% for women admitted during the delta period (table 4). Ten neonatal deaths occurred, five in the wildtype period, two in the alpha period, and three in the delta period. Overall, more than one in five babies were admitted for neonatal care (21.6%), with a trend towards increased risk in those born to mothers admitted during the alpha period compared with those admitted during the wildtype period (21.7% *v* 19.0% (adjusted odds ratio 1.24, 95% confidence interval 1.02 to 1.51)).

**Table 4:** Perinatal outcomes for babies of women admitted to UK hospitals with SARS-CoV-2 related symptoms during periods in which the original wildtype, alpha variant, and delta variant were dominant

Perinatal outcomes	Wildtype period (n=1358)*	Alpha period (n=1549)	Delta period (n=971)	Alpha *v* wildtype period	
				Odds ratio (95% CI)	Adjusted odds ratio (95% CI)†
Stillbirth	13 (1.0)	17 (1.1)	29 (3.0)	1.15 (0.58 to 2.25)	1.09 (0.54 to 2.20)
Admission to neonatal unit	254 (19.0)	332 (21.7)	250 (26.7)	1.21 (1.00 to 1.46)	1.24 (1.02 to 1.51)
Neonatal death	5 (0.4)	2 (0.1)	3 (0.3)	Not compared	Not compared

Data are number (%).

*Two women with singleton pregnancies known to have given birth but lost to follow-up and were excluded from the denominator of this column.

†Adjusted for age, ethnic origin, body mass index, employment, and presence of one or more pre-existing relevant medical comorbidity.

## Discussion

### Principal findings

This national prospective cohort study has identified that, after adjusting for personal characteristics and pre-existing medical conditions, the proportion of pregnant women admitted to UK hospitals with symptoms of covid-19 who experienced moderate to severe infection increased significantly from 25% to 36% and 43% in the periods when the original wildtype, alpha variant, and delta variant of SARS-CoV-2 were dominant, respectively. Pregnant women admitted with covid-19 in the alpha period were more likely to require respiratory support, have pneumonia, and be admitted to intensive care than those admitted in the wildtype period, with even greater risks in the delta period. While the majority of babies were live born, those born to mothers admitted in the alpha period were more likely to require admission for neonatal care than those born in the wildtype period. We purposely did not compare neonatal outcomes between delta and other periods because a high proportion of pregnancies were continuing at the time of analysis, as anticipated given the recent time frame of data collection. However, the available data suggest that stillbirths during this period might be increased.

### Strengths and limitations of this study

This national prospective cohort study compared pregnancy and perinatal outcomes by time period according to different dominant SARS-CoV-2 variants. A key strength of these data was the existing mechanism for national case identification of all women admitted to hospital across the UK, and therefore the low risk of selection bias. In the UK, universal SARS-CoV-2 testing for all obstetric admissions was implemented from May 2020, and a high proportion (37%) of pregnant women admitted to hospital with confirmed SARS-CoV-2 are asymptomatic and detected during screening on admission, most commonly to give birth.^16^ Therefore, while restricting the study to women with symptomatic infection introduces an unmeasurable but likely small risk of recall bias, overall this approach is a strength of the study because women presenting to hospital are inherently more likely to have an adverse outcome than the general pregnant population. Thus, the inclusion of all women testing positive for SARS-CoV-2, irrespective of symptom status, could result in increased adverse outcomes being incorrectly attributed to SARS-CoV-2 rather than misclassification bias, which affects most non-population based studies.^17^ Although a study limitation was that women with mild infection diagnosed and treated in the community were not included in this study, it is highly likely that all women with severe infection were captured in this analysis.

A further limitation of our study is that variant sequencing data were not available for individual women, and therefore proxy time periods were used instead. However, the delta variant is known to have contributed more than 90% of all sequenced cases since 7 June 2021 until the emergence of the omicron variant in December 2021, so major contamination is unlikely.^18^ Other time dependent changes will exist that we cannot account for—for example, varying thresholds for admission to hospital or intensive care depending on clinician familiarity with managing covid-19. In the general population, national guidance was updated in January 2021 to inform community management of those with oxygen saturations >92%.^19^ However, it is unclear whether this admission threshold was used extensively in pregnancy, and given that the Royal College of Obstetricians and Gynaecologists has never released national admission guidance for pregnant patients, this factor is unlikely to account for differences observed. Differing thresholds based on bed capacity might have been a contributory factor during the peak of the alpha variant wave when hospital pressures might have restricted admission to patients with the most severe disease. However, this restriction is not supported by our finding of an increased proportion of admissions primarily for covid-19 in the alpha period compared with the wildtype period.

In addition, hospital pressures from covid-19 (delta variant dominant) were not reported to be as high as during the second wave,^20^ which could explain the greater proportion of women admitted for covid-19 in this period, but it does not explain the increase in severe outcomes observed in this study. Similarly, the understanding of SARS-CoV-2 will also have improved over time. Publications during the wildtype period highlighted the increased risk of covid-19 in pregnancy in ethnic minority groups^21^—national guidance also focused on this and advised active health seeking in these groups.^22^ Should this guidance have resulted in a reduced threshold for admission then, contrary to our results, a reduced proportion of severe disease would have been observed over time.

### Comparison with other studies

The second rapid report from the MBRRACE-UK Confidential Enquiry into Covid-related Maternal Deaths, published in July 2021, emphasised the importance of considering additional risks associated with pregnancy in community escalation protocols,^23^ and hence also suggested a need for lower thresholds for admission. Data from the COPS (covid-19 in pregnancy in Scotland) study show that, from August 2020 when community testing was widely available, hospital admission rates as a proportion of total numbers of covid-19 infection in pregnancy were higher in both alpha dominance and delta dominance periods than the wildtype dominance period.^24^ These data therefore suggest that our findings of increased severity cannot be explained by higher thresholds for admission in alpha and delta periods, because overall admission rates (as a proportion of total infections) were higher in those periods than in the wildtype period, indicating either a lower threshold for admission or a greater severity of disease, or both.

We have reported a potential change in the proportion of pregnant women in paid employment between periods. This change could be the result of increased unemployment at the time, or an increased proportion of women from more deprived socioeconomic backgrounds, which might also explain the slightly increased proportion of smokers and younger women in this period. A limitation of this study was that further information on socioeconomic circumstances could not be collected owing to ethics committee requirements. This limitation is important when considering whether disease severity during different time periods might be attributed to the variant because the variant also affects disease transmission. For example, the alpha variant has been shown to have a higher secondary attack rate and therefore factors that increase transmission, such as multi-occupancy housing and public facing occupations, are important.^4^ Socioeconomic deprivation is also a known independent risk factor for adverse pregnancy outcomes, so this could be a source of residual confounding in this study.

Covid-19 specific drug treatments, which are now standard care, were used infrequently, even for women who were critically unwell. Based on the interim report from the RECOVERY trial, the Royal College of Obstetricians and Gynaecologists recommended in June 2020 that corticosteroid treatment should be considered for all women whose condition was clinically deteriorating.^22^ Despite restriction of this analysis to women admitted after 1 July 2021, steroid use remained low at 11.9% during the delta period, and while steroid use doubled in those critically unwell (28.7% during the delta period), this level still represents a small proportion of pregnant women being treated appropriately with drugs with long established use in pregnancy. In this study, 22 maternal deaths occurred, against a background of about 70 maternal deaths each year from all causes across the UK during or up to six weeks after pregnancy.^23^ The recent confidential enquiry (MBRRACE-UK) into care of all pregnant and postnatal women who died with SARS-CoV-2 infection found that only one in 10 had received treatment in accordance with the evidence based guidance, with most pregnant women receiving effective treatment and multidisciplinary maternity team involvement too late or not at all.^23^

Vaccination for all pregnant women regardless of risk group in the UK was recommended by the Joint Committee on Vaccination and Immunisation on 16 April 2021.^25^ Before this recommendation, vaccination had been available to pregnant women with underlying health conditions or increased risk of exposure since 31 December 2020.^26^ National data from Scotland suggest that vaccine uptake in pregnancy is very low, with 2% of the 3603 women who gave birth in May 2021 having any vaccine dose.^27^ Public Health England have also recently reported that of 51 724 pregnant women in England who have received their first dose so far, 20 648 had received both doses, compared with a population of 643 000 women giving birth each year in the UK.^25^ In our study, very few fully vaccinated pregnant women were admitted with symptomatic SARS-CoV-2, and the proportion with moderate or severe infection was reduced. A survey undertaken by the Royal College of Obstetricians and Gynaecologists in May 2021 reported that of 844 pregnant women offered vaccination, 58% had declined, mainly because of fear over safety for the mother and baby.^28^ There has been widespread misinformation regarding the impacts of the vaccination in young women.^29^ The findings of this study strongly highlight the urgent need for an international approach to tackle this misinformation and improve uptake of the vaccine during pregnancy, which is of even greater importance as infection continues to rapidly rise in both high and low resourced settings.^30^

### Conclusions

This national study has shown that pregnant women admitted during the periods in which the alpha and delta variants of SARS-CoV-2 were dominant were at increased risk of moderate to severe covid-19 infection, resulting in admission to intensive care, compared with those admitted during the period when the original wildtype was dominant. Research on the effects of infection during the omicron period on pregnant women, and its association with vaccination status, is now imperative because rates could differ again. Pregnant women with covid-19 are already known to be at an increased risk of admission to intensive care compared to those without infection, irrespective of the time period.^16^ Further follow-up is required to clarify the risk of infection during the delta dominant period on perinatal outcomes such as stillbirth. Effective treatments are now available, but are used in only a minority of women, even among those who are critically unwell. Our vaccine data support the effectiveness of immunisation in pregnancy, yet vaccine uptake is reported to be low compared with the general population. Urgent action to tackle misinformation and policy change to prioritise actions to promote uptake are required, given the increasing rates of infection nationally and internationally.^31^ Future research is required to identify the characteristics associated with severe infection and vaccine uptake across risk groups in pregnancy, in order to inform future policy.
