# Supplementary material for: Severity of maternal infection and perinatal outcomes during periods of SARS-CoV-2 wildtype, alpha, and delta variant dominance in the UK: prospective cohort study
Source: BMJ Med. 2022 Feb 28;1(1):e000053. doi: 10.1136/bmjmed-2021-000053 (PMC9978672; doi:10.1136/bmjmed-2021-000053)
Supplement: Supplementary data [file bmjmed-2021-000053supp001.pdf]

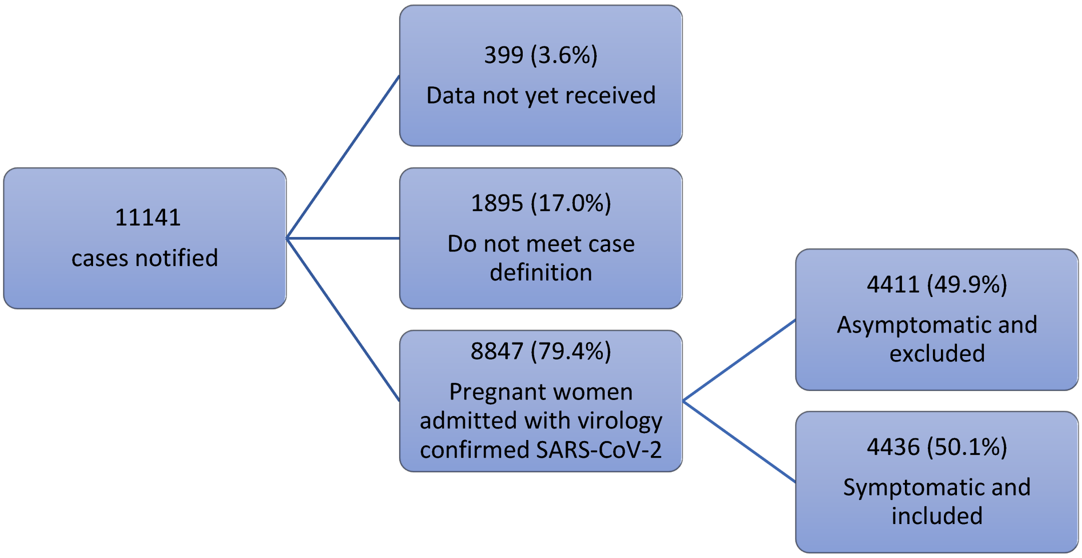

Supplementary Figure 1: Women included in study from 1st March 2020 to 31st October 2021

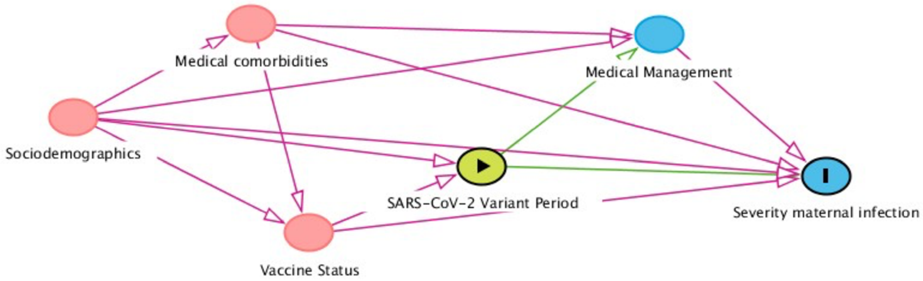

Supplementary Figure 2: Directed acyclic graphs indicating covariates and mediators on relationship between SARS-CoV-2 variant and severity of maternal infection.
