# Supplementary material for: Severity of maternal infection and perinatal outcomes during periods of SARS-CoV-2 wildtype, alpha, and delta variant dominance in the UK: prospective cohort study
Source: BMJ Med. 2022 Feb 28;1(1):e000053. doi: 10.1136/bmjmed-2021-000053 (PMC9978672; doi:10.1136/bmjmed-2021-000053)
Supplement: Supplementary data [file bmjmed-2021-000053supp002.pdf]

SUPPLEMENTARY TABLES

Supplementary Table 1: Respiratory and medical support of pregnant women symptomatic of SARS-CoV-2 during the periods in which the Wildtype and Alpha variants were dominant (Adjusted odds ratios from multiple imputation by chained equation (MICE) models)

	**aOR Alpha vs. Wildtype (95% CI)***	**aOR Delta vs. Wildtype (95% CI)***
**Composite indicator of moderate to severe infection**	1.99 (1.65-2.39)	2.58 (2.15-3.10)
**Oxygen saturation measured on admission (Yes)**	5.05 (3.97-6.42)	7.21 (5.56-9.36)
Oxygen saturation <95%	1.63 (1.16-2.30)	1.54 (1.05-2.26)
**Evidence of pneumonia on imaging**	1.88 (1.51-2.35)	2.42 (1.98-2.97)
**Respiratory support required**	2.11 (1.70-2.64)	2.66 (2.11-3.35)
Non-invasive oxygen (nasal canulae, mask or non-rebreathe mask at <15l/min)	REF	REF
High flow oxygen (>15l/min) or CPAP	0.73 (0.45-1.16)	1.10 (0.69-1.76)
Invasive Ventilation or ECMO	0.62 (0.41-0.94)	0.67 (0.44-1.03)
**Intensive Care admission**	1.66 (1.31-2.10)	2.47 (1.87-3.26)
**Pharmacological Management Total****	2.60 (1.91-3.53)	2.43 (1.77-3.33)

*Adjusted for age, ethnicity, body mass index (BMI), employment and presence of one or more pre-existing relevant medical comorbidity.
** Any of the listed medications given for medical management of SARS-CoV-2: antivirals, Tocilizumab, maternal steroids, monoclonal antibodies.
CPAP = continuous positive airway pressure. ECMO = extracorporeal membrane oxygenation. REF = reference group against which other categories were compared.

Supplementary Table 2: Pregnancy outcomes for women symptomatic of SARS-CoV-2 during the periods in which the Wildtype and Alpha variants were dominant (Adjusted odds ratios from multiple imputation by chained equation (MICE) models)

Pregnancy outcomes	aOR Alpha vs. Wildtype (95% CI)*
**Gestation at birth (weeks)**	
<22	NC
22-27^{+6}	2.17 (1.09- 4.29)
28-34^{+6}	1.29 (0.96- 1.73)
34-36^{+6}	1.05 (0.83- 1.32)
37 or more	REF
Median (IQR)***	-0.10 (-0.29- 0.08)
**Delivery expedited due to COVID-19****	1.63 (1.25- 2.14)
**Mode of birth****	
Pre-labour Caesarean	1.20 (1.01- 1.43)
Caesarean after labour onset	0.95 (0.75- 1.19)
Operative vaginal	0.85 (0.65- 1.11)
Unassisted vaginal	REF

*Adjusted for age, ethnicity, body mass index (BMI), employment and presence of one or more pre-existing relevant medical comorbidity
** Excluding pregnancy loss from denominator. NC=not compared. REF = reference group against which other categories were compared.
***Quantile (median) regression with robust and clustered standard errors

Supplementary Table 3: Perinatal outcomes for women symptomatic of SARS-CoV-2 during the periods in which the Wildtype and Alpha variants were dominant (Adjusted odds ratios from multiple imputation by chained equation (MICE) models)

Perinatal outcomes	aOR Alpha vs. Wildtype (95% CI)*
**Stillbirth**	1.12 (0.57-2.20)
**Admission to Neonatal Unit**	1.19 (0.98-1.44)

*Adjusted for age, ethnicity, body mass index (BMI), employment and presence of one or more pre-existing relevant medical comorbidity
Two women with singleton pregnancies known to have given birth but lost to follow up and were excluded from the denominator of Wildtype.

Supplementary Table 4: Respiratory and medical support of pregnant women symptomatic of SARS-CoV-2 during the periods in which the Wildtype and Alpha variants were dominant (Table 2 results but BMI and age modelled as restricted cubic spline functions with three knots located at BMI values of 21.3, 27.3, and 37.6 and ages 23 years, 31 years, and 38 years, respectively, instead of categorical)

	**aOR Alpha vs. Wildtype (95% CI)***	**aOR Delta vs. Wildtype (95% CI)***
**Composite indicator of moderate to severe infection**	1.95 (1.63-2.33)	2.65 (2.20-3.19)
**Oxygen saturation measured on admission (Yes)**	5.30 (4.42-6.37)	7.45 (6.09-9.12)
Oxygen saturation <95%	1.59 (1.11-2.29)	1.57 (1.09-2.28)
**Evidence of pneumonia on imaging**	1.82 (1.50-2.22)	2.51 (2.05-3.07)
**Respiratory support required**	1.40 (1.13-1.74)	1.89 (1.52-2.36)
Non-invasive oxygen (nasal canulae, mask or non-rebreathe mask at <15l/min)	REF	REF
High flow oxygen (>15l/min) or CPAP	0.79 (0.48-1.32)	1.13 (0.68-1.88)
Invasive Ventilation or ECMO	0.73 (0.47-1.16)	0.75 (0.47-1.20)
**Intensive Care admission**	1.81 (1.37-2.38)	2.71 (2.06-3.57)
**Pharmacological Management Total****	2.68 (2.04-3.53)	2.52 (1.89-3.35)

*Adjusted for age, ethnicity, body mass index (BMI), employment and presence of one or more pre-existing relevant medical comorbidity.
** Any of the listed medications given for medical management of SARS-CoV-2: antivirals, Tocilizumab, maternal steroids, monoclonal antibodies.
CPAP = continuous positive airway pressure. ECMO = extracorporeal membrane oxygenation. REF = reference group against which other categories were compared.

Supplementary Table 5: Pregnancy outcomes for women symptomatic of SARS-CoV-2 during the periods in which the Wildtype and Alpha variants were dominant (Table 3 results but BMI and age modelled as restricted cubic spline functions with three knots located at BMI values of 21.3, 27.3, and 37.6 and ages 23 years, 31 years, and 38 years, respectively, instead of categorical)

**Pregnancy outcomes**	**aOR Alpha vs. Wildtype (95% CI)***
**Gestation at birth (weeks)**	
<22	NC
22-27^{+6}	2.44 (1.15- 5.14)
28-34^{+6}	1.35 (1.00- 1.85)
34-36^{+6}	1.05 (0.83- 1.33)
37 or more	REF
Median (IQR)***	-0.12 (-0.30- 0.06)
**Delivery expedited due to COVID-19****	1.04 (0.77- 1.40)
**Mode of birth****	
Pre-labour Caesarean	1.15 (0.96- 1.38)
Caesarean after labour onset	0.91 (0.71- 1.15)
Operative vaginal	0.81 (0.62- 1.06)
Unassisted vaginal	REF

*Adjusted for age, ethnicity, body mass index (BMI), employment and presence of one or more pre-existing relevant medical comorbidity
** Excluding pregnancy loss from denominator.
***Quantile (median) regression using bootstrap sampling
NC=not compared. REF = reference group against which other categories were compared.

Supplementary Table 6: Perinatal outcomes for women symptomatic of SARS-CoV-2 during the periods in which the Wildtype and Alpha variants were dominant (Table 4 results but BMI and age modelled as restricted cubic spline functions with three knots located at BMI values of 21.3, 27.3, and 37.6 and ages 23 years, 31 years, and 38 years, respectively, instead of categorical)

**Perinatal outcomes**	**aOR Alpha vs. Wildtype (95% CI)***
**Stillbirth**	1.10 (0.55-2.22)
**Admission to Neonatal Unit**	1.25 (1.02-1.53)

*Adjusted for age, ethnicity, body mass index (BMI), employment and presence of one or more pre-existing relevant medical comorbidity
Two women with singleton pregnancies known to have given birth but lost to follow up and were excluded from the denominator of Wildtype.
